# Engineering the Assembly Freedom of Donor–Acceptor Type Self‐Assembled Monolayers Toward Efficient and Stable Flexible Perovskite Photovoltaics

**DOI:** 10.1002/adma.74486

**Published:** 2026-08-05

**Authors:** Biao Zhou, Guosen Zhang, Hao Wang, Juntao Zhao, Ruijia Zhang, Hong Zhang, Mingwei An, Yue Wang, Yang Wang, Wallace C. H. Choy

**Affiliations:** ^1^ Department of Electrical and Electronic Engineering The University of Hong Kong Hong Kong Hong Kong SAR China; ^2^ Strait Institute of Flexible Electronics (SIFE, Future Technologies) Fujian Key Laboratory of Flexible Electronics Fujian Normal University Fuzhou Fujian China; ^3^ State Key Laboratory of Ultra‐intense Laser Science and Technology Shanghai Institute of Optics and Fine Mechanics (SIOM) Chinese Academy of Sciences (CAS) Shanghai China; ^4^ State Key Laboratory of Photovoltaic Science and Technology Fudan University Shanghai China

**Keywords:** assembly freedom, carrier extraction, flexible perovskite solar cells, interfacial strain dissipation, mechanical robustness, self‐assembled monolayers

## Abstract

Flexible perovskite solar cells (f‐PSCs) are promising contenders for portable and wearable photovoltaics, yet developing f‐PSCs that simultaneously achieve high power conversion efficiency (PCE) and superior operational stability, including mechanical robustness, remains challenging. Herein, we propose a strategy of modulating the assembly freedom of donor–acceptor type self‐assembled monolayers (SAMs) to address this issue. The newly designed 2FMPA‐BT‐PPA (PPA) SAMs exhibit higher assembly freedom on flexible ITO substrates compared to the previously reported 2FMPA‐BT‐BA (BA) SAMs, delivering two key benefits: first, they enable the formation of a higher‐quality monolayer via improved conformational adaptability and strengthened π–π stacking, which facilitates efficient carrier transport. Second, the SAMs’ conformational adaptability and uniformly tilted orientations can effectively dissipate interfacial strain under external mechanical loads, thereby enhancing the mechanical robustness of flexible devices. These synergies yield f‐PSCs with a champion PCE of 25.3% (26.3% for rigid device), alongside exceptional operational stability. Crucially, PPA‐based devices retain 98% initial PCE after 10 000 multidirectional bending cycles (3 mm radius) with no observable structural damage, outperforming BA‐based devices and all reported SAMs‐based f‐PSCs. This work offers donor‐acceptor SAMs design experiences for efficient, robust f‐PSCs, revealing assembly freedom's key role in interfacial carrier extraction and mechanical robustness.

## Graphical abstract

Assembly freedom engineering breaks the vertical alignment constraint of donor–acceptor self‐assembled monolayers, tuning their assembly freedom to achieve quasi‐random molecular orientation. This strategy simultaneously boosts interfacial charge transport and mechanical robustness, enabling high‐efficiency and mechanically stable flexible perovskite solar cells.

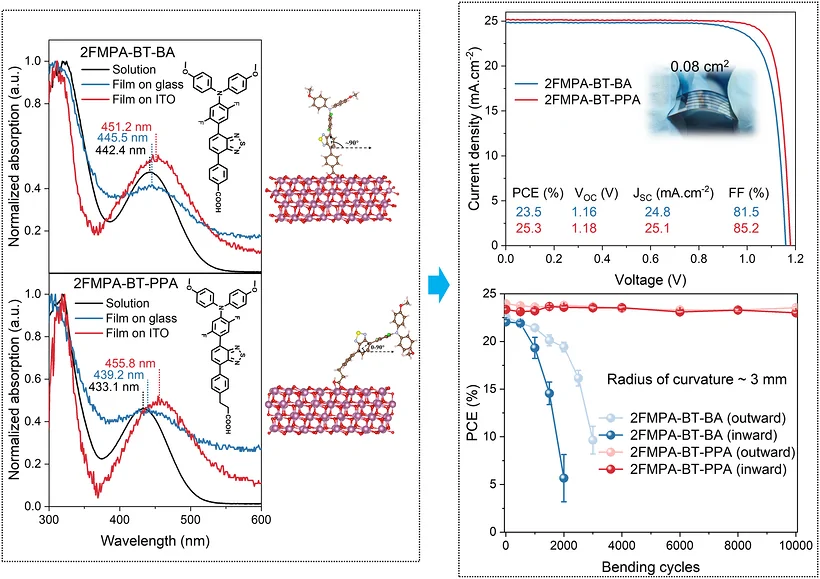

## 1 Introduction

Perovskite solar cells (PSCs) represent the most rapidly advancing photovoltaic technology, with a certified power conversion efficiency (PCE) exceeding 27% [1, 2, 3]. The pursuit of lightweight, flexible, and high‐efficiency photovoltaics has positioned PSCs as a frontrunner for next‐generation wearable and portable power sources [4]. However, the practical deployment of flexible perovskite solar cells (f‐PSCs) is severely constrained by the long‐standing trade‐off between high photovoltaic performance and operational stability, particularly the mechanical robustness under repeated bending deformation, which remains far inferior to that of rigid counterparts [5, 6, 7]. A core bottleneck lies in the rational design of the interfacial layer between the flexible transparent conductive substrate (typically polyethylene naphthalate/indium tin oxide, PEN/ITO) and the perovskite photoactive layer [5, 8]. Among various interfacial materials, self‐assembled monolayers (SAMs) have emerged as a superior candidate owing to their ultra‐thin thickness, tunable energy levels, strong chemical anchoring to substrates, and multifunctional capabilities in interfacial defect passivation and perovskite crystallization regulation [9, 10, 11]. To date, carbazole‐based SAMs are the most widely reported for high‐performance rigid PSCs and f‐PSCs [12, 13, 14, 15, 16, 17, 18].

Donor–acceptor (D–A) type SAMs, featuring a tailor‐made electron‐donating (D) unit and electron–accepting (A) unit in the conjugated backbone, have recently emerged as a versatile alternative to conventional carbazole‐based SAMs [19, 20]. Benefiting from their abundant functionalization sites and highly tunable optoelectronic properties, D–A SAMs enable synergistic regulation of interfacial dipole engineering, defect passivation, and charge separation/extraction, thus delivering impressive PCEs in rigid PSCs [21, 22, 23, 24, 25]. Nevertheless, research on D–A SAMs remains far less extensive than that on carbazole‐based counterparts, and more critically, few studies have systematically investigated the rational design of D–A SAMs specifically for f‐PSCs. Unlike rigid glass/ITO substrates, flexible PEN/ITO substrates undergo continuous interfacial strain during repeated bending deformation, which can readily trigger desorption of SAMs, disruption of molecular stacking, and deterioration of interfacial contact [5, 6, 26]. These issues additionally induce perovskite film cracking, interfacial delamination, and catastrophic device performance degradation. Meanwhile, existing studies on D–A SAMs mainly focus on modifying the functional groups of D and A moieties to improve the optoelectronic performance of rigid devices, while completely neglecting the critical impact of the molecular assembly freedom of SAMs on their assembly behavior on flexible substrates, interfacial mechanical adaptability, and the long‐term mechanical robustness of f‐PSCs. This critical oversight leaves a key research gap in developing D–A SAMs that can simultaneously achieve high efficiency and exceptional mechanical durability for state‐of‐the‐art f‐PSCs.

Herein, we report a rational molecular design strategy to modulate the assembly freedom of D–A SAMs via linker‐anchoring unit length tuning, targeting the development of efficient and mechanically robust f‐PSCs. Building on our previously reported D–A SAMs, 2FMPA‐BT‐BA [21] (denoted as BA), where benzoic acid acts as the linker‐anchoring moiety, we designed a structural analog, 2FMPA‐BT‐PPA (denoted as PPA), in which the benzoic acid moiety is replaced by phenylpropionic acid to extend the alkyl linker segment. This structural modification endows PPA with significantly higher assembly freedom on PEN/ITO substrates, bringing two key performance enhancements. First, PPA enables more efficient interfacial charge extraction than BA via strengthened π–π stacking, as its enhanced conformational adaptability better accommodates the rough ITO surface, forming a denser, more ordered monolayer for better carrier transport. Second, in addition to improving interfacial adhesion and crystallinity of perovskite, the PPA monolayer efficiently dissipates interfacial strain accumulated during mechanical bending, suppressing interfacial degradation. This behavior stems from the uniformly tilted molecular stacking and conformational adaptability afforded by its high assembly freedom, whereas BA's limited assembly freedom causes inhomogeneous stacking that fails to relieve strain, leading to severe interfacial failure. Benefiting from these merits, PPA‐based f‐PSCs achieve a champion PCE of 25.3% for 0.08 cm^2^ devices and 23.4% for 1.00 cm^2^ devices (26.3% for the rigid device), outperforming BA‐based devices. After 1400 h of continuous maximum power point tracking (MPPT) testing, PPA‐based devices retain 92% of their initial PCE, compared with 81% for BA‐based devices. Most notably, PPA‐based devices maintain over 98% of their initial PCE after 10 000 bending cycles at a 3 mm radius, with no observable perovskite cracking or interfacial delamination; in contrast, BA‐based devices retain less than 50% of their initial PCE after fewer bending cycles, accompanied by severe SAMs desorption, perovskite cracking, and interfacial failure. Additionally, PPA‐based f‐PSCs not only achieve one of the highest reported V_OC_ × FF values among SAMs‐based f‐PSCs, but also exhibit superior mechanical robustness relative to all previously reported SAMs‐based f‐PSCs. This work offers design insight for D–A SAMs targeting efficient and mechanically robust f‐PSCs, and reveals the critical role of molecular assembly freedom in regulating interfacial carrier extraction and mechanical durability for flexible optoelectronic devices.

## 2 Results and Discussion

### 2.1 SAMs’ Assembly Freedom, Molecular Stacking, and Interfacial Properties

For D–A SAMs with a linker between the anchoring group and the conjugated backbone, we propose “assembly freedom” as a conceptual framework to describe the ability of the conjugated backbone segment to undergo conformational and orientational adjustments relative to the substrate surface after the anchoring group has covalently bound to the substrate. It is not a single directly measurable physical quantity, but an intrinsic attribute of the molecular assembly system that can be quantitatively evaluated and cross‐verified through multiple complementary experimental metrics. This conformational flexibility allows adjacent molecules to optimize their relative orientations and distances to achieve stronger intermolecular π‐π stacking interactions and more ordered monolayer structures. We hypothesize that the linker length of SAMs can exert profound effects on their assembly freedom, which could further affect molecular stacking, interfacial charge transport dynamics, and mechanical strain dissipation in f‑PSCs. We reasoned that a longer linker would enhance conformational adaptability, enabling denser, more ordered molecular stacking and uniform orientation distribution on flexible PEN/ITO substrates. This should promote efficient charge extraction and suppress non‑radiative recombination, while also dissipating mechanical strain at the interface during repeated bending. Guided by these rationales, we designed the PPA molecule on the previously developed BA molecule. The inset of Figure 1a shows the structure of the two molecules; they share identical D and A segments, allowing us to exclusively reveal the influence of linker length on assembly freedom without interference from optoelectronic variations in the D‐‑A backbone. The material synthetic details are provided in Note S1.

**FIGURE 1:**
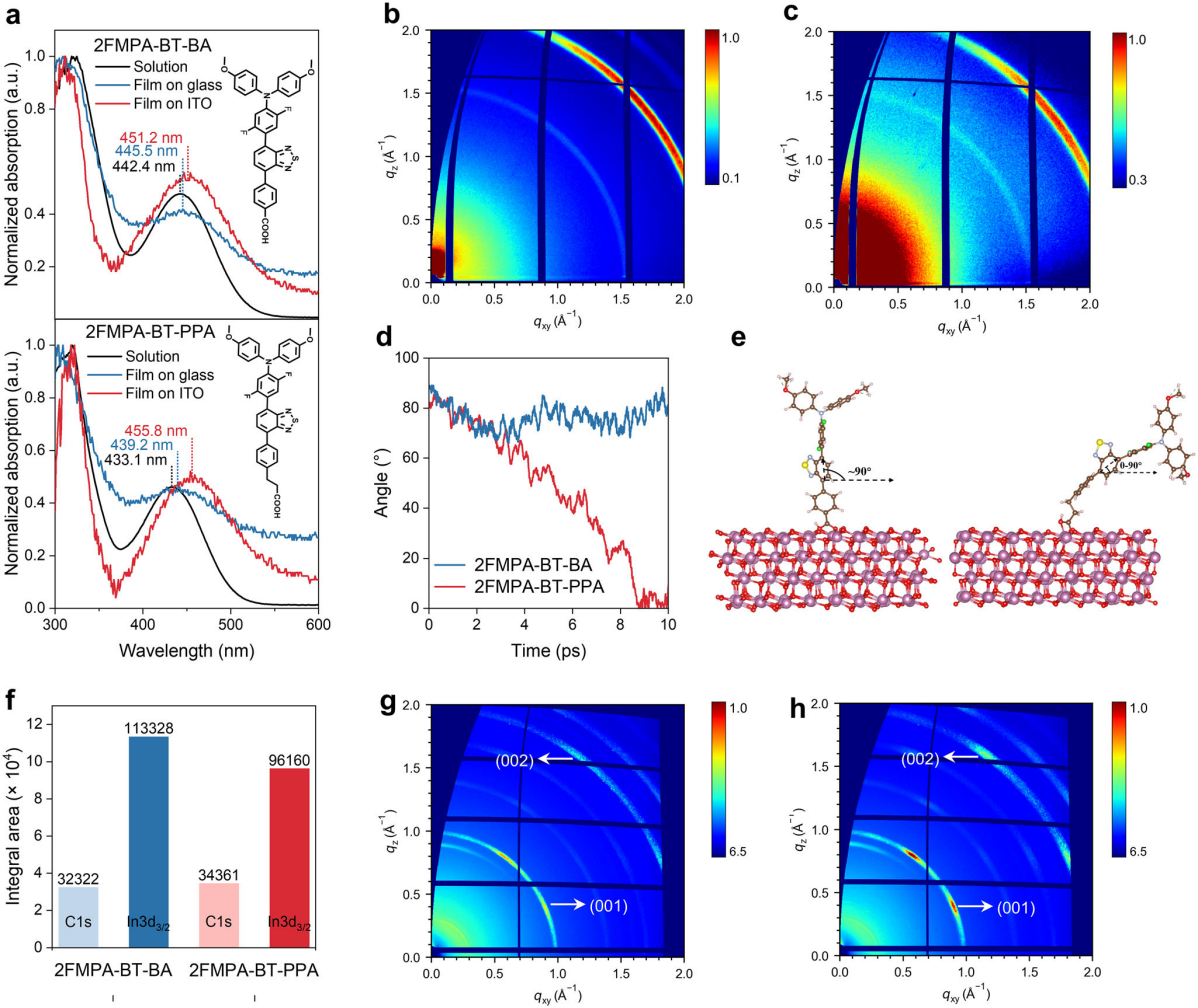
SAMs’ assembly freedom, molecular stacking, and interfacial properties. (a) Normalized UV–absorption spectra of 2FMPA‐BT‐BA (up) and 2FMPA‐BT‐PPA (down) in solution state, film state on glass and film state on PEN/ITO. (b,c) Two‐dimensional GIWAXS patterns for PEN/ITO/2FMPA‐BT‐BA (b) and PEN/ITO/2FMPA‐BT‐PPA (c). (d) Angular evolution of D–A SAMs (2FMPA‐BT‐BA and 2FMPA‐BT‐PPA) on ITO (111) surface during AIMD simulations at 300 K. (e) A representative intermediate snapshot of 2FMPA‐BT‐BA (left) SAMs and 2FMPA‐BT‐PPA SAMs (right) on ITO (111) surface from AIMD trajectory. The pink, red, brown, yellow, green, and blue spheres represent In, O, C, S, F, and N atoms, respectively. (f) The C1s and In_3_d_3/2_ integral area of PEN/ITO/2FMPA‐BT‐BA and PEN/ITO/2FMPA‐BT‐PPA. (g,h) Two‐dimensional GIWAXS patterns for perovskite films deposited on PEN/ITO/2FMPA‐BT‐BA (g) and on PEN/ITO/2FMPA‐BT‐PPA (h).

To quantitatively evaluate the assembly freedom of D–A SAMs and its direct correlation with intermolecular π–π stacking interactions, we performed UV–vis absorption spectroscopy measurements under three well‐defined conditions. Figure 1a presents the absorption spectra of BA and PPA in dilute solution, as films on glass substrates, and as covalently anchored SAMs on PEN/ITO substrates. In dilute solution (10^−5^ M), where intermolecular interactions are negligible, BA and PPA exhibit absorption peaks at 442.4 and 433.1 nm, respectively. These values serve as a baseline reference for the intrinsic electronic structure of individual molecules, consistent with their identical conjugated backbones. When deposited on glass substrates (non‐anchored aggregated state), both molecules show red‐shifted absorption peaks due to the formation of *J*‐type π–π stacking [27]. BA exhibits a small red‐shift of 3.1 nm (442.4 to 445.5 nm), while PPA shows a significantly larger red‐shift of 6.1 nm (433.1 to 439.2 nm). Since the two molecules differ only in alkyl linker length, this difference directly demonstrates that the longer propyl linker in PPA confers greater intrinsic conformational flexibility, allowing molecules to adjust their conjugated segments to achieve stronger intermolecular interactions even in the absence of covalent anchoring. On PEN/ITO substrates (covalently anchored self‐assembled state), the difference in assembly freedom becomes even more pronounced. BA exhibits a red‐shift of 8.8 nm (442.4 to 451.2 nm), while PPA shows a dramatic red‐shift of 22.7 nm (433.1 to 455.8 nm). This result reveals that after covalent binding to the substrate, PPA molecules still retain sufficient conformational freedom to optimize intermolecular stacking, whereas BA molecules with the shorter linker are conformationally constrained and cannot form equivalent strong π–π interactions. These absorption spectroscopy results show that PPA exhibits a significantly larger redshift of the absorption peak after anchoring on the substrate, which directly quantifies the stronger intermolecular π–π stacking in PPA SAMs. Since stronger π–π stacking requires molecules to retain sufficient conformational flexibility to optimize intermolecular positions after covalent anchoring, this experimental observation serves as a primary quantitative metric to evaluate the difference in assembly freedom between BA and PPA. This confirms that linker length modulation effectively tunes the assembly behavior of D–A SAMs, which in turn governs their intermolecular stacking and monolayer structural quality.

To further elucidate the origin of the enhanced intermolecular π–π stacking interactions, we performed grazing‐incidence wide‐angle x‐ray scattering (GIWAXS) measurements to characterize the molecular assembly and orientation of BA and PPA SAMs on flexible PEN/ITO substrates (Figure 1b,c). Both BA and PPA SAMs exhibited characteristic scattering arcs centered at *q*_xy_ ≈ 1.53 Å^−1^, which corresponds to the intermolecular π–π stacking distance of the conjugated backbones [15], spanning a broad azimuthal range. Notably, the BA SAMs displayed a sharp, discrete diffraction spot at an azimuthal angle of ∼0°, indicating a predominantly vertical molecular alignment with limited conformational tilting. In stark contrast, the PPA SAMs showed no distinct preferential diffraction spots across the entire 0°–90° azimuthal range, instead exhibiting a uniformly distributed scattering intensity along the π–π stacking arc. This experimental observation confirms that PPA molecules adopt a uniformly tilted orientation spanning 0°–90° on the PEN/ITO surface, whereas BA exhibits a predominantly vertical alignment with limited tilting. This broad orientational distribution aligns well with the expected behavior of higher assembly freedom: molecules with greater conformational flexibility can adopt more diverse stable configurations after anchoring, resulting in a uniformly tilted orientation distribution. The ability of PPA molecules to freely adjust the tilt angle of their conjugated backbones both before and after covalent anchoring to the substrate allows for more optimal intermolecular packing and stronger π–π orbital overlap. Consistently, the PPA SAMs exhibited significantly higher overall scattering intensity in the π–π stacking region compared to the BA SAMs, which is in excellent agreement with the larger absorption red‐shift observed in UV–vis spectroscopy measurements. These results collectively demonstrate that the uniformly tilted orientation enabled by greater assembly freedom effectively promotes stronger intermolecular π–π stacking interactions. A recent study has shown that this uniformly tilted orientation of SAMs is beneficial for interfacial hole extraction [28].

To further validate the experimental observations of molecular orientation, we performed ab initio molecular dynamics (AIMD) simulations to investigate the dynamic adsorption behavior and conformational adaptability of BA and PPA on the ITO (111) surface. Adsorption models of the two molecules on the ITO (111) surface were first constructed and structurally relaxed, followed by simulations at 300 K for 10 ps with a 1 fs time step (10 000 total iterations). A computational method is provided in Note S2. We defined the orientation angle as the angle between the central benzene ring of each molecule and the ITO surface normal (*z*‐axis), and statistically analyzed its temporal evolution (Figure 1d). Figure 1e displays the representative intermediate snapshot of BA and PPA on the ITO surface from the AIMD trajectory. The orientation angle evolution reveals that BA, anchored to the ITO surface via a rigid single‐bond linkage, maintains an orientation angle near 90° (nearly perpendicular to the surface) throughout the simulation with minimal fluctuations, reflecting limited conformational freedom. In stark contrast, PPA exhibits substantially higher interfacial flexibility due to its extended linker, enabling significant molecular conformational distortion and a broad distribution of orientation angles spanning 0°–90°. The simulation results are generally in good agreement with our GIWAXS observations: BA adopts a predominantly perpendicular orientation at equilibrium, while PPA shows no preferred orientation, with angles uniformly distributed across 0°–90°. The wider range of orientation angle fluctuations at the atomic scale provides theoretical quantitative support for the difference in conformational flexibility, which is consistent with the higher assembly freedom of PPA inferred from experimental results. This distinct behavior arises directly from the rigid single‐bond anchoring of BA, which restricts molecular reorientation, whereas the extended linker of PPA enhances assembly freedom, enabling diverse molecular configurations on the flexible substrate.

Building on the insights into molecular orientation and stacking from the above analysis, we further characterized the self‐assembly quality, interfacial properties, and charge transport capabilities of BA and PPA on PEN/ITO substrates. The relative surface coverage of SAMs was calculated from the ratio of C1s to In_3_d_3/2_ peak areas in XPS spectra (Figure 1f; full spectra provided in Figure S9), revealing that PPA exhibited a coverage factor of 10.2 × 10^−3^, 18.6% higher than that of BA (8.6 × 10^−3^). Energy‐dispersive x‐ray spectroscopy (EDS) further qualitatively confirmed higher fluorine loading on PEN/ITO/PPA than PEN/ITO/BA (Figure S10, Tables S1 and S2), consistent with the denser self‐assembly of PPA. Integrating UV–vis absorption spectroscopy and GIWAXS analysis, the enhanced assembly freedom of PPA eliminates the steric constraints that prevent BA molecules from packing closely. This is directly confirmed by our XPS and EDS measurements, which show a significantly higher surface coverage factor for PPA SAMs compared to BA SAMs. This improved monolayer quality is a logical consequence of enhanced assembly freedom: molecules with greater conformational flexibility can pack more closely without steric hindrance. In contrast, the rigid vertical alignment of BA molecules leads to larger intermolecular distances and a looser, more defective monolayer structure.

Kelvin probe force microscopy (KPFM) measurements (Figure S11) showed that PEN/ITO/PPA exhibited substantially reduced surface potential inhomogeneity compared to PEN/ITO/BA (samples’ corresponding atomic force microscope imaging is shown in Figure S12), which facilitates uniform and efficient interfacial charge extraction. This is a direct experimental observation of more uniform interfacial electrical properties, which arises from the denser and more homogeneous PPA SAMs coverage. Given that enhanced assembly freedom enables molecules to pack more closely and uniformly without steric constraints, this improved potential uniformity provides indirect supporting evidence for the higher assembly freedom of PPA SAMs. Scanning electron microscopy (SEM) imaging (Figure S13) revealed a clearer surface morphology for PEN/ITO/PPA, reflecting the higher conductivity and improved hole‐extraction capability. The improved electrical conductivity of the SAMs interlayers originates primarily from their denser and more uniform surface coverage. Nano‐scratch measurements (Figure S14) were performed to assess the interfacial adhesion strength between the perovskite and PEN/ITO/SAMs, revealing that PEN/ITO/PPA (3233 µN) exhibited 59% higher adhesion strength than PEN/ITO/BA (2029 µN), likely attributed to the denser PPA SAMs structure enabling more robust and uniform interfacial interactions (e.g., favorable fluorine‐perovskite bonding). As the improved monolayer density and uniformity are key consequences of enhanced assembly freedom, this result further corroborates the validity of our assembly freedom design framework from the perspective of mechanical interface properties.

To establish the mechanistic link between SAMs assembly behavior and device performance, we systematically characterized the interfacial carrier dynamics and energetic alignment using complementary techniques. Transient absorption spectroscopy (TAS) reveals faster ground‐state bleach recovery for PPA‐based heterostructures (Figure S15), directly verifying accelerated hole extraction from perovskite to ITO through the PPA interlayer; this trend is further supported by TRPL and steady‐state PL measurements (Figures S16 and S17). To identify the origin of this improvement, we constructed the interfacial energy level diagram based on DFT calculations, UV–vis absorption, and ultraviolet photoelectron spectroscopy (UPS) of exfoliated perovskite bottom surfaces (Figures S18–S21). Both SAMs exhibit well‐aligned HOMO levels with the perovskite valence band maximum, ruling out energy level alignment differences as the primary cause of the performance gap. Photoluminescence quantum yield (PLQY) measurements show a 50% higher PLQY for perovskite films on PPA SAMs (Figure S22), confirming suppressed non‐radiative recombination at the buried interface. Device‐level transient photocurrent/transient photovoltage (TPC/TPV) measurements are consistent with these thin‐film results: PPA‐based devices show faster photocurrent decay and slower photovoltage decay (Figure S28), verifying both enhanced charge collection and reduced recombination at the full device scale. Collectively, these results rule out energetic alignment differences as the primary cause of the performance gap, and instead suggest that the superior interfacial optoelectronic performance of PPA SAMs arises from the higher‐quality monolayer structure enabled by enhanced assembly freedom, which reduces interfacial traps and accelerates charge transfer.

Generally, these characterizations demonstrate that the increased assembly freedom of PPA, which enhances molecular conformational adaptability on the rough PEN/ITO substrate, strengthens π–π stacking interactions and enables the formation of denser, more ordered SAM. These structural advantages simultaneously improve interfacial electrical transport, physical contact uniformity, and mechanical adhesion, laying a critical foundation for both efficient and mechanically robust f‐PSCs.

Finally, the GIWAXS results in Figure 1g,h demonstrate that perovskite films deposited on PEN/ITO/PPA exhibit markedly enhanced diffraction intensity and stronger preferential (001) crystal plane orientation compared to those on PEN/ITO/BA, indicating a substantial improvement in perovskite crystallinity. Space‐charge‐limited current (SCLC) measurements (Figure S23) reveal that the defect density of perovskite films on PEN/ITO/PPA is reduced by over 55% relative to BA‐based counterparts. Collectively, these results demonstrate that the improved crystalline quality of perovskite films on PEN/ITO/PPA can be attributed to the denser and more uniform SAMs formed by PPA molecules. This enhanced SAMs quality reduces bottom‐interface defects via more effective passivation (e.g., fluorine‐mediated passivation of uncoordinated lead ions in perovskite) and mitigates nucleation and crystallization inhomogeneity of the overlying perovskite film.

### 2.2 Interfacial Strain Dissipation Enabled by SAMs

Interfacial strain characterization reveals that PPA SAMs effectively mitigate interfacial strain accumulation during cyclic mechanical bending, which is critical for enhancing the mechanical robustness of flexible devices. This strain‐buffering behavior correlates closely with the uniformly tilted molecular orientation of PPA, as the greater conformational flexibility enables dynamic structural adjustment under mechanical deformation.

To investigate the strain dissipation capability of the SAMs, we mechanically exfoliated the perovskite layer from the PEN/ITO/SAMs/perovskite stacks and conducted grazing‐incidence x‐ray diffraction (GIXRD) on the perovskite bottom interface to characterize interfacial strain. For fresh samples, GIXRD patterns (Figure S24) show that perovskite diffraction peaks shift slightly toward lower angles with increasing instrument angle, regardless of whether the film is deposited on PEN/ITO/BA or PEN/ITO/PPA. This behavior indicates mild tensile strain at both bottom interfaces, consistent with previous reports [29]. Stark differences emerge after cyclic mechanical bending aging (3000 inward bending cycles at a curvature radius of 3 mm). For the aged BA sample (Figure 2a), perovskite diffraction peaks shift significantly toward lower angles with increasing instrument angle, indicating severe tensile strain accumulation at the bottom interface due to strain concentration. In contrast, the aged PPA sample (Figure 2b) shows a diffraction peak shift nearly identical to that of the fresh PPA sample, with only minor low‐angle shifts as the instrument angle increases, confirming that no significant strain accumulation occurs at the interface. To quantify strain evolution, we constructed 2*θ*‐sin^2^ *φ* plots from the GIXRD patterns (Figure 2c), where strain magnitude is proportional to the absolute slope of the fit line. The BA sample shows a ∼2.5‐fold increase in interfacial strain after aging, while the PPA sample exhibits only a 17% strain increase. These results confirm that PPA SAMs act as an effective interfacial strain buffer layer between PEN/ITO and perovskite, capable of dissipating mechanical strain during cyclic bending, whereas BA fails to mitigate strain accumulation at the interface.

**FIGURE 2:**
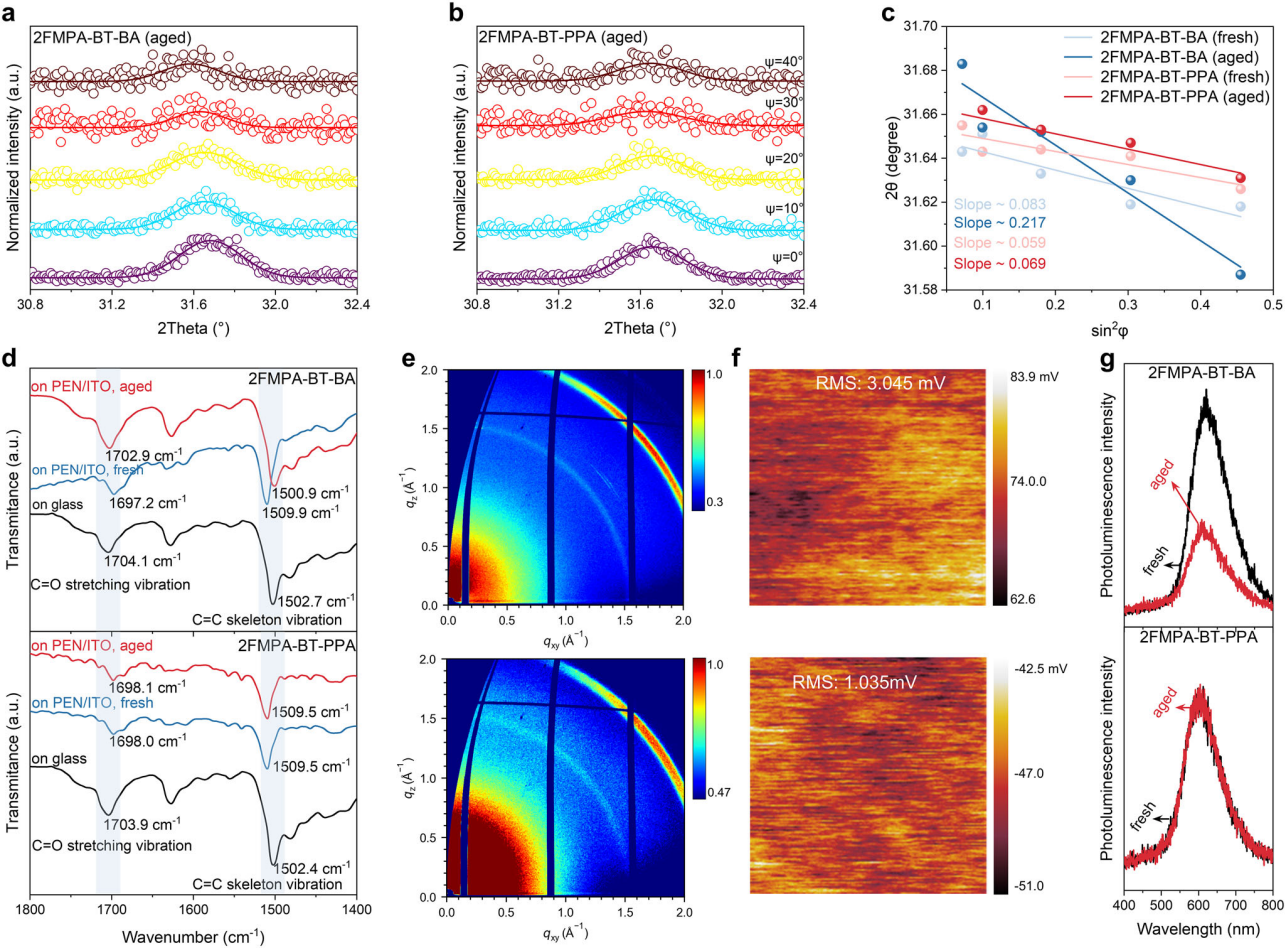
Interfacial strain dissipation enabled by SAMs. (a,b) GIXRD diffraction patterns of the bottom surface of perovskite films peeled from PEN/ITO/2FMPA‐BT‐BA/perovskite sample (a) and PEN/ITO/2FMPA‐BT‐PPA/perovskite sample (b) after mechanical bending cycle aging, with instrument angles from 0° to 40°. (c) Calculation of the relative magnitude of macroscopic strain for the fresh and mechanically aged perovskite films from PEN/ITO/D‐A SAMs/perovskite samples by classical 2*θ*‐sin^2^ *φ* method. (d) The FTIR spectra of glass/D‐A SAMs, PEN/ITO/ D‐A SAMs, and PEN/ITO/D‐A SAMs with mechanical aging; 2FMPA‐BT‐BA (up) and 2FMPA‐BT‐PPA (down). (e) Two‐dimensional GIWAXS patterns for PEN/ITO/2FMPA‐BT‐BA after mechanical aging (up) and PEN/ITO/2FMPA‐BT‐PPA after mechanical aging (down). (f) The potential mapping of PEN/ITO/2FMPA‐BT‐BA (up) and PEN/ITO/2FMPA‐BT‐PPA (down) after mechanical aging measured by KPFM. The area of the tested region is 3 µm × 3 µm. (g) The PL spectra of PEN/ITO/2FMPA‐BT‐BA (up) and PEN/ITO/2FMPA‐BT‐PPA (down) before and after mechanical aging.

To uncover the molecular‐level mechanism underlying the distinct strain dissipation behaviors, we first conducted Fourier‐transform infrared (FTIR) spectroscopy to characterize the anchoring interactions between SAMs and ITO, as well as intermolecular conjugated π‐stacking, before and after cyclic mechanical bending aging (Figure 2d). For BA: when anchored on PEN/ITO, the carboxyl group's coordination with ITO weakens the C═O bond strength, red‐shifting its vibrational frequency from 1704.1 cm^−1^ (glass‐deposited films) to 1697.2 cm^−1^. Concurrently, intermolecular π‐conjugated stacking in the SAMs strengthens the conjugated backbone C═C bond, blue‐shifting its vibrational frequency from 1502.7 cm^−1^ (glass‐deposited films) to 1509.9 cm^−1^, consistent with our absorption spectroscopy results (Figure 1a). For mechanically aged BA SAMs on PEN/ITO, the C═O vibrational frequency blue‐shifts back to 1702.9 cm^−1^, indicating the disruption of carboxyl‐ITO anchoring interactions. Concurrently, the conjugated C═C bond frequency red‐shifts to 1500.9 cm^−1^, reflecting weakened intermolecular π‐stacking. These results confirm that mechanical aging induces BA desorption from ITO, accompanied by degraded intermolecular conjugated interactions. For PPA: on PEN/ITO, the C═O frequency red‐shifts from 1703.9 cm^−1^ (glass‐deposited films) to 1698.0 cm^−1^ due to carboxyl‐ITO coordination, while the conjugated C═C frequency blue‐shifts from 1502.4 to 1509.5 cm^−1^ due to enhanced self‐assembled π‐stacking. Notably, mechanically aged PPA SAMs retain a C═O frequency of 1698.1 cm^−1^ and a C═C frequency of 1509.5 cm^−1^, nearly identical to fresh samples. This confirms that PPA SAMs maintains stable anchoring to ITO and intact intermolecular conjugated stacking after mechanical aging.

To further validate these findings, we conducted GIWAXS on mechanically aged PEN/ITO/SAMs samples to characterize molecular stacking evolution (Figure 2e). Compared to fresh BA SAMs (Figure 1b), aged BA SAMs lose the 0° azimuth diffraction spot at *q*_xy_ ≈ 1.53 Å^−1^ (corresponding to vertically oriented molecules), while the scattering arc from tilted molecules remains. This confirms that the dominant vertically oriented BA fraction desorbs severely during aging, leaving only the minor tilted fraction intact. In stark contrast, aged PPA SAMs retain a scattering arc nearly identical to fresh samples (Figure 1c), confirming that the uniformly tilted PPA SAMs remain fully anchored to ITO without desorption.

Collectively, these results reveal the origin of the distinct mechanical durability: BA's limited assembly freedom enforces a rigid, predominantly vertical orientation on ITO, which prevents conformational adjustment under mechanical stress, leading to strain concentration, SAMs desorption, and degraded intermolecular π‐stacking. In contrast, PPA's high assembly freedom enables a uniformly tilted, flexible molecular arrangement that dissipates strain via conformational adjustment, avoiding strain accumulation and maintaining stable ITO anchoring and intermolecular π‐stacking. These conclusions are corroborated by KPFM surface potential mapping of aged samples (Figure 2f): PEN/ITO/BA shows drastically reduced potential uniformity due to desorption‐induced molecular disorder (compared to fresh samples in Figure S11), while PEN/ITO/PPA retains uniform potential distribution identical to fresh samples. Steady‐state PL measurements of PEN/ITO/SAMs (Figure 2g) further confirm this trend: BA exhibits a significant PL intensity reduction after aging, while PPA shows unchanged PL intensity. These findings thus establish a robust structure–property correlation between SAMs’ assembly freedom, interfacial strain dissipation capability, and mechanical robustness on flexible PEN/ITO substrates, laying a critical molecular foundation for the outstanding mechanical durability exhibited by PPA‐based f‐PSCs under cyclic bending conditions.

### 2.3 The Enhanced Photovoltaic Performance and Stability of f‐PSCs

Building on the superior interfacial properties of PPA SAMs demonstrated above, we fabricated p‐i‐n inverted f‐PSCs with the architecture: PEN/ITO/SAMs (BA or PPA)/perovskite/PEABr/C_60_/BCP/Ag (PEABr = phenethylammonium bromide; BCP = bathocuproine) to evaluate their photovoltaic performance. For small‐area devices (0.08 cm^2^), the champion BA‐based f‐PSC delivers a PCE of 23.5%, with an open‐circuit voltage (*V*_OC_) of 1.16 V, short‐circuit current density (*J*_SC_) of 24.8 mA cm^−2^, and fill factor (FF) of 81.5%. In contrast, the champion PPA‐based small‐area device achieves a higher PCE of 25.3%, with a *V*_OC_ of 1.18 V, *J*_SC_ of 25.1 mA cm^−2^, and FF of 85.2% (Figure 3a). The *J*_SC_ values derived from *J*–*V* measurements are in excellent agreement with integrated current densities calculated from external quantum efficiency (EQE) spectra (Figure S25). Steady‐state maximum power output measurements over 300 s demonstrate that BA‐based devices maintain a steady PCE of 22.9%, while PPA‐based devices retain 25.1% (Figure S26), consistent with *J*–*V* measurements.

**FIGURE 3:**
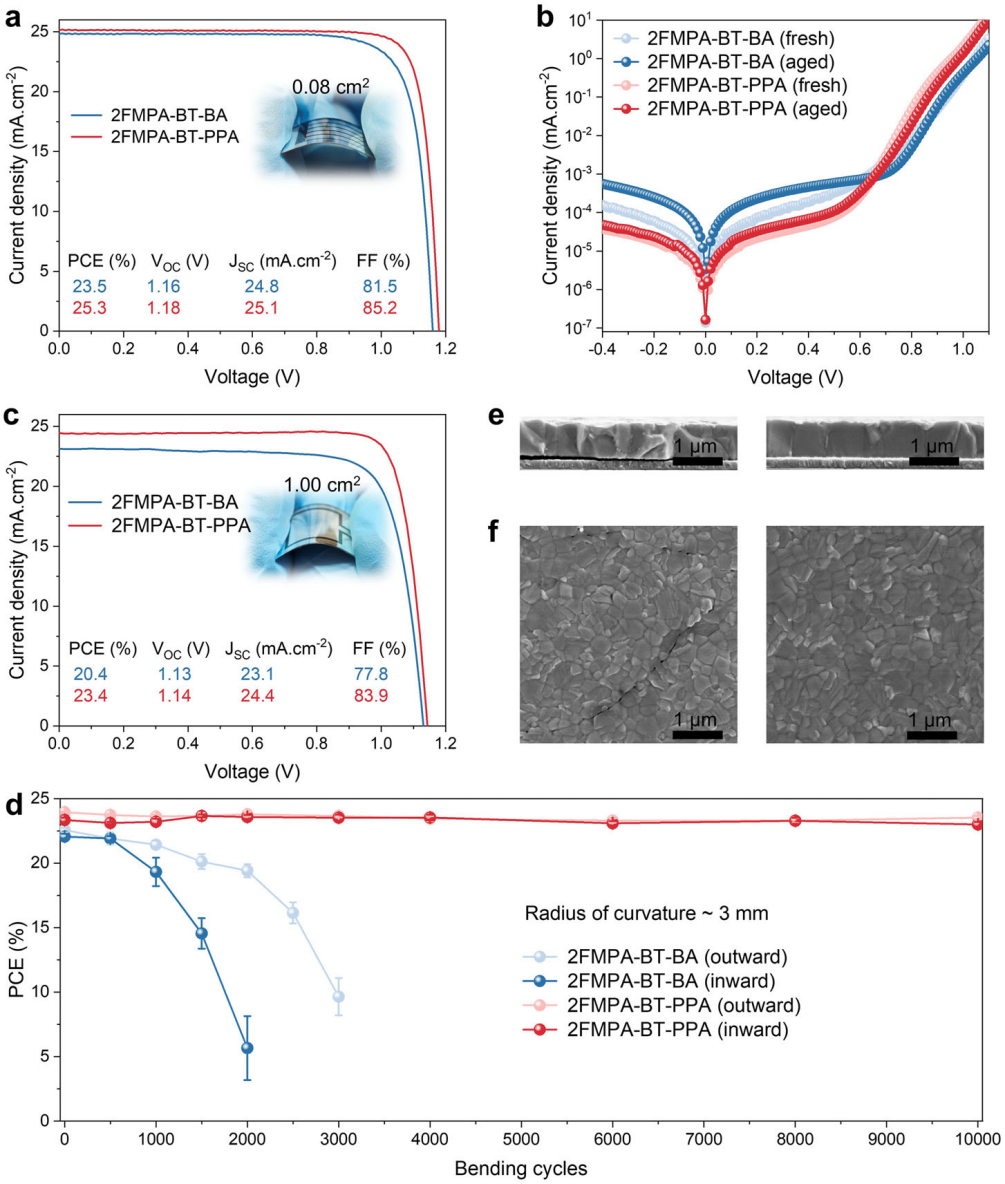
The enhanced photovoltaic performance and stability of f‐PSCs. (a) The champion *J*–*V* curves of the f‐PSCs under reverse scans (device area ∼ 0.08 cm^2^). (b) The dark *J*–*V* curves of the f‐PSCs before and after mechanical bending cycle aging. (c) The champion *J*–*V* curves of the f‐PSCs under reverse voltage scans (device area ∼ 1.00 cm^2^). (d) Performance evolution of f‐PSCs as a function of multidirectional mechanical bending cycles at a bending radius of 3 mm. (e) The cross‐sectional morphology of the PEN/ITO/2FMPA‐BT‐BA/perovskite (left) after 2000 inward bending cycles at a 3 mm curvature radius and of the PEN/ITO/2FMPA‐BT‐PPA/perovskite (right) after 10000 inward bending cycles at a 3 mm curvature radius. (f) The surface morphology of the PEN/ITO/2FMPA‐BT‐BA/perovskite (left) after 2000 inward bending cycles at a 3 mm curvature radius and of the PEN/ITO/2FMPA‐BT‐PPA/perovskite (right) after 10 000 inward bending cycles at a 3 mm curvature radius.

Statistical performance distribution (Figure S27) confirms that PPA‐based devices exhibit consistently higher PCEs than BA‐based devices (24.77% ± 0.23% vs. 23.03% ± 0.26%). The performance gains primarily originate from the markedly enhanced FF (84.31% ± 0.51% vs. 81.78% ± 0.68%) and *V*_OC_ (1.175 ± 0.003 V vs. 1.152 ± 0.007 V). TPC and TPV measurements indicate that PPA‐based devices exhibit faster photocurrent decay and slower photovoltage decay compared to BA‐based devices (Figure S28). Therefore, the improvements in FF and *V*_OC_ are attributed to the dense, ordered PPA SAMs endowed by their higher assembly freedom, which enables stronger π–π stacking interactions, uniformly tilted molecular orientations and higher surface coverage, thereby optimizing intimate interfacial contact, facilitating efficient charge extraction, and suppressing interfacial non‐radiative recombination. This conclusion is further corroborated by the lower dark current density of PPA‐based devices under reverse bias (Figure 3b). Forward and reverse *J*–*V* scans (Figure S29) reveal negligible hysteresis in both devices. To evaluate scalability, we fabricated large‐area flexible devices (1.00 cm^2^ active area): the PPA‐based device achieves a PCE of 23.4% with an FF of 83.9%, whereas the BA‐based device only reaches 20.4% with an FF of 77.8% (Figure 3c). For reference, we also fabricated rigid PSCs on ITO glass: the champion PPA‐based rigid device delivers a PCE of 26.3%, while the BA‐based rigid device achieves 24.5% (Figure S30 and Table S3).

To examine the transferability of our assembly freedom engineering strategy across D‐A SAMs with different conjugated backbone modifications, we further investigated a secondary D–A system with a monofluorinated backbone, FMPA‐BT‐BA, and FMPA‐BT‐PPA. As shown in Figure S31, UV–vis absorption measurements reveal that the absorption peak of FMPA‐BT‐BA redshifts by 5.6 nm from the solution state (451.0 nm) to the ITO‐anchored film state (456.6 nm), while FMPA‐BT‐PPA with the extended linker exhibits a larger redshift of 8.6 nm (from 440.0 nm in solution to 448.6 nm on ITO). This consistent trend of enhanced redshift with extended linker length indicates that stronger intermolecular π–π stacking can be achieved by increasing conformational flexibility, a behavior that is not exclusive to the difluorinated backbone in the main system. Consistent with the improved molecular packing, FMPA‐BT‐PPA‐based f‐PSCs deliver superior photovoltaic performance compared to the FMPA‐BT‐BA control (Figure S32). The champion FMPA‐BT‐PPA device achieves a PCE of 24.3%, with an average PCE of 24.01% ± 0.24% across five independent devices, along with a champion *V*_OC_ of 1.17 V (average: 1.164 ± 0.004 V) and FF of 83.9% (average: 83.63% ± 0.42%). In contrast, the FMPA‐BT‐BA control devices yield a champion PCE of 22.8% (average: 22.62% ± 0.16%), with a *V*_OC_ of 1.16 V (average: 1.158 ± 0.003 V) and FF of 81.4% (average: 80.89% ± 0.79%). The performance improvement is dominated by an enhanced FF and a moderately increased *V*_OC_, which follows the same trend observed in the main difluorinated system. These preliminary results provide initial support for a transferable molecular design principle: for D–A type SAMs, moderate extension of the linker to enhance assembly freedom represents a broadly applicable optimization strategy to improve monolayer quality and photovoltaic performance.

To assess the mechanical robustness of the f‐PSCs under practical deformation conditions, we performed multi‐directional (inward and outward) cyclic bending tests with a fixed bending curvature radius of 3 mm. The details of the cyclic bending test are provided in the Characterization Section and Figure S33. Figure 3d shows that BA‐based devices exhibit severe performance degradation starting from 500 bending cycles, retaining only 22.1% and 43.2% of their initial PCE after 2000 inward and 3000 outward bending cycles, respectively. In stark contrast, PPA‐based devices retain over 98.5% and 98.2% of their initial PCE even after 10 000 inward and outward bending cycles, demonstrating drastically enhanced mechanical durability. This stark performance discrepancy can be attributed to the synergistic effects of multiple interfacial improvements, among which the enhanced interfacial strain dissipation capability conferred by PPA's high conformational freedom likely plays a dominant role. The improved mechanical robustness also benefits from three key complementary factors: (1) significantly enhanced interfacial adhesion energy (as confirmed by nano‐scratch tests of Figure S14); (2) more homogeneous surface chemistry provided by the denser and more uniform PPA SAM, which reduces crack initiation sites (as confirmed by XPS tests of Figure 1f); and (3) improved perovskite crystallization inherently enhances the mechanical flexibility of the perovskite film (as confirmed by GIWAXS tests of Figure 1g,h). Specifically, the uniformly tilted orientation of PPA molecules allows them to dynamically adjust their tilt angles to accommodate mechanical deformation, effectively dissipating interfacial strain and preventing microcrack propagation. In contrast, the rigid vertically aligned BA molecules have limited conformational flexibility, leading to strain accumulation and rapid interfacial degradation under repeated bending cycles.

To unveil the origin of this mechanical stability, we performed cross‐sectional and surface morphology characterization via SEM on PEN/ITO/SAMs/perovskite stacks after 2000 inward bending cycles (BA) and 10 000 inward bending cycles (PPA). For the BA sample, SEM images (Figure 3e,f) reveal severe interfacial delamination between the ITO/BA layer and perovskite film, along with obvious cracks on the perovskite surface. This failure is closely associated with BA's limited assembly and conformational freedom, which hinders effective strain dissipation. Based on the FTIR and GIWAXS results of aged SAMs, we infer that mechanical stress induces BA desorption from the ITO surface, weakening interfacial bonding and triggering delamination, while unrelieved strain concentration propagates into the perovskite film and causes cracking. In contrast, SEM characterization of the PPA sample shows intimate contact between the ITO/PPA layer and the perovskite film, with no observable cracks on the perovskite surface after 10 000 bending cycles. This exceptional mechanical robustness stems from PPA's superior strain dissipation capability: the conformational adaptability brought by high assembly freedom enables PPA to remain firmly anchored to ITO and maintain strong interfacial interactions with perovskite, effectively relieving mechanical stress and suppressing both delamination and perovskite cracking.

To further verify the interfacial integrity after mechanical aging, we performed dark *J*–*V* measurements on BA‐based devices (after 2000 inward bending cycles) and PPA‐based devices (after 10 000 inward bending cycles). As shown in Figure 3b, the dark current density of aged BA devices is significantly higher than that of fresh devices, whereas the dark current of aged PPA devices remains nearly identical to that of their fresh counterparts. This result indicates that BA's inability to dissipate strain leads to severe interfacial degradation and perovskite damage, while PPA's strain dissipation capability effectively preserves interfacial integrity and perovskite film quality. In addition, we evaluated the mechanical durability of two additional D–A‐type SAM molecules, previously reported by us [19, 23], that share the same benzoic acid linker as the BA molecule. The results are shown in Figure S34. The PCE of f‐PSCs based on these two SAMs was lower, and both exhibited a significant decline in performance similar to that of the BA molecule after 2000 outward bending cycles at a bending radius of 3 mm. This further confirms that PPA SAMs offer unique advantages in simultaneously enhancing the optoelectronic performance and mechanical durability of f‐PSCs. Table S4 summarizes recently reported efficient SAMs‐based f‐PSCs and their corresponding mechanical robustness performance metrics. Our PPA‐based f‐PSCs not only achieve among the highest reported *V*_OC_ × FF values, a key indicator of balanced interfacial charge extraction and transport, but also exhibit superior mechanical durability compared to all previously reported SAMs‐based f‐PSCs. This dual superiority highlights the efficacy of our assembly freedom modulation strategy in simultaneously optimizing photovoltaic performance and mechanical durability for flexible perovskite devices, thus addressing a longstanding trade‐off between these two critical performance parameters in f‐PSCs.

Beyond mechanical robustness, we further evaluated the long‐term operational stability of the f‐PSCs under accelerated aging conditions via MPPT measurements. As shown in Figure S35, after 1400 h of continuous MPPT operation under ambient atmosphere (55°C, 60% relative humidity), BA‐based device retains 81.3% of their initial PCE, while PPA‐based device maintains 92.2% of their initial PCE. This enhanced operational stability of PPA‐based devices further corroborates the structural and interfacial advantages of PPA SAMs: the dense, ordered self‐assembled structure not only facilitates efficient charge extraction but also effectively isolates the perovskite active layer from ambient moisture and oxygen, suppressing non‐radiative recombination and device degradation.

## 3 Conclusions

In summary, this work demonstrates a rational assembly freedom modulation strategy of D–A SAMs for realizing efficient and mechanically robust f‐PSCs. By extending the linker‐anchoring moiety of the reference BA SAMs to yield the PPA SAMs, we enhanced the assembly freedom of the SAMs and thereby improved their conformational adaptability on PEN/ITO substrates. This improvement enables denser molecular stacking and uniform tilted orientations, which facilitate interfacial carrier transport and interfacial strain dissipation. The PPA‐based f‐PSCs achieved a champion PCE of 25.3% for 0.08 cm^2^ devices and 23.4% for 1.00 cm^2^ devices, outperforming BA‐based counterparts. Notably, PPA devices retained 98% of initial PCE after 10 000 multidirectional bending cycles at 3 mm radius and 92% of initial PCE after 1400 h of MPPT operation, while BA devices suffered severe performance decay. Our PPA‐based f‐PSCs also rank among the top‐performing SAM‐based f‐PSCs reported to date, with both superior *V*_OC_ × FF metrics and unmatched mechanical robustness. Based on the comprehensive characterization results, these performance improvements can be mainly attributed to the high assembly freedom of PPA SAMs, which enhances charge extraction, suppresses non‐radiative recombination, improves interfacial adhesion and perovskite crystallinity, and efficiently dissipates interfacial strain. This work reveals the critical role of SAMs’ assembly freedom in regulating the interfacial properties of flexible optoelectronics, providing an insightful experience for achieving efficient and stable f‐PSCs.

## Conflicts of Interest

The authors declare no conflicts of interest.

## Supporting information




**Supporting File**: adma74486‐sup‐0001‐SuppMat.docx.

## Data Availability

The data that support the findings of this study are available from the corresponding author upon reasonable request.
