# Supplementary material for: Engineering the Assembly Freedom of Donor–Acceptor Type Self‐Assembled Monolayers Toward Efficient and Stable Flexible Perovskite Photovoltaics
Source: Adv Mater. 2026 Aug 5;38(56):e74486. doi: 10.1002/adma.74486 (PMC13648892; doi:10.1002/adma.74486)
Supplement: Supplementary file 1 [file ADMA-38-e74486-s001.docx]

Supplementary Information for

**Engineering the Assembly Freedom of Donor-Acceptor Type Self-assembled Monolayers Towards Efficient and Stable Flexible Perovskite Photovoltaics**

Biao Zhou *et al.*

Corresponding author: Wallace C.H. Choy, chchoy@eee.hku.hk;

Yang Wang, ifewangy@fjnu.edu.cn

**This PDF file includes:**

1. Experimental section

2. Characterization section

3. Materials synthesis (Note S1)

4. Computation method (Note S2)

5. Figure S9 to Figure S33

6. Table S1 to Table S4

7. References

**Experimental section**

Materials: All chemicals and solvents were used as received without further purification. Formamidine iodide (FAI) and methylammonium iodide (MAI) were purchased from GreatCell Solar (Australia). Cesium iodide (CsI), methylammonium Chloride (MACl), phenethyl ammonium bromide (PEABr), and poly(bis(4-phenyl)(2,4,6- trimethylphenyl) amine) (PTAA) were purchased from Xian Polymer Light Technology Corp (China). Lead iodide (PbI_2_) and [2-(9H-Carbazol-9-yl) ethyl] phosphonic acid (2PACz) were purchased from TCI (Japan). Isopropanol (IPA), dimethylformamide (DMF, 99.8%), dimethyl sulfoxide (DMSO), and chlorobenzene (CB) were purchased from Sigma-Aldrich (USA). Phenyl-C61-butyric acid methyl ester (PCBM) was purchased from Advanced Election Co,. Ltd. (China). Bathocuproine (BCP) was purchased from Acros Organics (USA). Silver Pellets were purchased from Kurt J. Lesker (USA). PEN/ITO flexible electrodes were purchased from Shangyang Technology (China). The base and curing agent of polydimethylsiloxane (PDMS) were purchased from Dow Corning (USA).

Preparation of glass/PDMS stack: The PDMS solution was prepared by mixing 1 mL of PDMS base and 100 μL of PDMS curing agent in 2 mL of hexane. The resulting solution was then spin-coated onto a cleaned glass substrate at 6000 rpm for 30 seconds, followed by an annealing at 120 °C for 30 minutes to form the PDMS film. Here, PDMS served as an adhesive film, allowing the PEN/ITO flexible electrode to be attached to the glass/PDMS stack. This glass/PDMS stack functions as a supporting rigid substrate throughout the fabrication processes of f-PSCs.

Preparation of perovskite precursor solution: The 1.65 M perovskite precursor solution was prepared by mixing CsI, FAI, MAI, and PbI_2_ in DMF: DMSO (4:1/v: v) with a chemical formula of Cs_0.05_MA_0.1_FA_0.85_PbI_3_. MACl was added to the precursor to improve the crystallinity of the perovskite film.

Fabrication of f-PSCs on PEN/ITO substrate: Firstly, the PEN/ITO electrode was ultrasonically cleaned for 20 min using detergent, DI water, acetone, and ethanol, then the PEN/ITO electrode was treated with ultraviolet ozone (UVO) for 20 min. After that, 2FMPA-BT-BA or 2FMPA-BT-PPA solution (1.0 mg/mL in CB) was spin-coated on top of the PEN/ITO electrode at 4000 rpm for 30 s and was then annealed at 100 ℃ for 10 min. Then, the perovskite precursor solution was spin-coated on Glass/PDMS/PEN/ITO/SAMs at 1000 rpm for 6 s and then 5000 rpm for 30 s. 200 μL CB antisolvent was dropped onto the perovskite film at 14 s before the end of the second spin-coating step. The obtained wet perovskite films were annealed at 100 ℃ for 30 min. Passivator PEABr (1 mg/ml in IPA and DMSO solution, volume ratio 200:1) was dynamically spin-coated on the surface (4000 rpm, 30 s) of the perovskite film and annealed at 100 °C for 5 min. After that, PCBM (20mg/ml in CB) was spin-coated at 1500 rpm for 30 s and then annealed at 100℃ for 30 min to form the electron transport layer. Followed by BCP buffer layer deposition by dynamically spin-coating its solution (0.5mg/ml in IPA) at 4000 rpm for 30s. Finally, 100 nm silver was thermally evaporated under a high vacuum (< 6 × 10-6 Torr). The device area was defined by a metal shadow mask. After finishing all the fabrication processes, f-PSCs were peeled off from the Glass/PDMS rigid supporting substrates.

**Characterization Section**

GIWAXS was performed at BL03HB beamline of Shanghai Synchrotron Radiation Facility (SSRF) using the X-ray energy of 10 KeV. Two-dimensional images were acquired by a PLATUS 2M detector mounted vertically at a distance of ~104 mm from the sample with a grazing-incidence angle of 0.2° and an exposure time of 200 s. Ultraviolet photoelectron spectroscopy (UPS) measurements were conducted by ThermoFisher ESCALAB 250Xi. FTIR spectra were measured using a Fourier transform infrared spectrometer (JASCO, FT/IR-6600). UV-vis absorption spectra were obtained from a UV-vis spectrometer (Shimadzu, UV-2600). The XPS spectra were tested on an XPS spectrometer (ThermoFischer, ESCALAB Xi+) with an Al Kα X-ray source. AFM and KPFM measurements were conducted using an Atomic Force Microscope (NT-MDT NTEGRA). The PL and TRPL spectra were measured using a fluorescence spectrophotometer (Pico Qaunt Fluo Time 300) with excitation at 375 nm. Transient absorption spectrometry (TAS) was performed by TA100 (Time-tech Spectra, Dalian). The GIXRD patterns were acquired using a diffractometer equipped with CuKα radiation (λ = 1.5406 Å, Rigaku SmartLab). High-resolution SEM (Hitachi S-4800) with EDS analysis function was used to observe film morphology and composition. Electron distribution of HOMO/LUMO energy level of SAMs were calculated by density functional theory (DFT) with Gaussian09 package at a level of B3LYP/6-311G(d,p). The nano-scratch test was conducted using Bruker Ti77. The details of the AIMD simulation are provided in Note S2. Mechanical aging tests on f-PSCs were conducted using a home-made cyclic bending/folding machine.

J-V curves were measured by a Keithley 2400 source meter under 100 mW/cm^2^ (SS- F5- 3A, Enli Technology Co., Ltd.). A standard silicon solar cell (SRC-2020, Enli Technology Co., Ltd.) was used to calibrate the light intensity. EQE spectra were performed on a solar cell quantum efficiency measurement system (QE-R) provided by Enli Technology Co., Ltd. For TPC and TPV measurements, the devices were placed under steady-state illumination from a white LED to simulate one-sun working condition and the carriers were excited by a 550 nm pulse laser. TPC decay was recorded using a digital oscilloscope at open-circuit condition and TPC decay was recorded under approximately short-circuit condition with a 50 Ω resistor series connected to the device. Cyclic bending tests on f-PSCs were conducted using our home-made cyclic bending tester. The details of the cyclic bending test were provided in **Figure S31**.

**Materials synthesis (Note S1)**

All reagents and chemicals are commercially purchased and are used without further purification unless otherwise stated. Solvents were purified by standard methods and dried if necessary. 2FMPA-BT-BA was synthesized according to our previous work. Proton and carbon nuclear magnetic resonance spectra (^1^H NMR and ^13^C NMR) were characterized by a Bruker Advance III 400 (400 MHz for ^1^H and 100 MHz for ^13^C) NMR spectroscope.

Scheme S1. Synthesis of 2FMPA-BT-PPA.

**Compound 1**: A mixture of bis(4-methoxyphenyl)amine (458 mg, 2.0 mmol), 1,4 dibromo-2,5-difluorobenzene (813 mg, 3.0 mmol), Pd_2_(dba)3 (38 mg, 0.04 mmol), BINAP (50 mg, 0.08 mmol), sodium tert-butoxide (400 mg, 4.0 mmol) in o-xylene (15 mL) was heated to reflux overnight. After cooling to room temperature, the mixture was filtered and concentrated. The crude product was purified by column chromatography (silica gel, petroleum ether:CH_2_Cl_2_ = 5:1 as the eluent) to afford a light-yellow solid in 54% yield (454 mg). ^1^H NMR (400 MHz, CDCl_3_): δ 7.25-7.21 (m, 1H), 6.95 (d, J = 9.0 Hz, 1H), 6.83 (d, J = 9.0 Hz, 1H), 6.81-6.77 (m, 1H), 3.81 (s, 6H). ^19^F NMR (376 MHz, CDCl_3_): δ -111.97 (d, J = 14.1 Hz, 1F), -121.97 (d, J = 14.1 Hz, 1F)

**Compound 2**: A mixture of compound 1 (800 mg, 2 mmol), bis(pinacolato)diboron (762 mg, 3 mmol), Pd(dppf)Cl_2_ (73 mg, 0.1 mmol) and potassium acetate (588 mg, 6 mmol) in dioxane (30 mL) was heated at 110 ℃ for 18h. After cooling to room temperature, the mixture was filtered and concentrated. The crude product was purified by column chromatography (silica gel, petroleum ether:CH_2_Cl_2_ = 1:4 as the eluent) to afford a yellow solid in 73% yield (650 mg). ^1^H NMR (400 MHz, CDCl_3_): δ 7.37-7.34 (m, 1H), 6.97 (d, J = 9.0 Hz, 4H), 6.83 (d, J = 9.0 Hz, 4H), 6.64-6.60 (m, 1H), 3.81 (s, 6H), 1.36 (s, 12H). ^19^F NMR (376 MHz, CDCl_3_): δ -107.11 (d, J = 17.5 Hz, 1F), -125.35 (d, J = 17.6 Hz, 1F).

**Compound 3**: To a 25 mL dried flask was charged with 4,7-dibromobenzo[c][1,2,5]thiadiazole (400 mg, 1.36 mmol), (4-(3-ethoxy-3-oxopropyl)phenyl)boronic acid (302 mg, 1.36 mmol), Pd(PPh_3_)_4_ (47 mg, 0.04 mmol), K_2_CO_3_ (264 mg, 1.904 mmol), THF (10 mL), and water (1 mL), and heated to reflux for 12 h under nitrogen atmosphere. After cooling to room temperature, the reaction mixture was extracted with CH_2_Cl_2_ three times. The combined organic layer was dried over anhydrous Na_2_SO_4_. All reagents were removed, the residue was purified by column chromatography (silica gel, petroleum ether: CH_2_Cl_2_ = 2:1 as the eluent) to afford a light-yellow solid in a yield of 63% ( mg). ^1^H NMR (600 MHz, CDCl_3_): δ 7.92 (d, J = 7.6 Hz, 1H), 7.82 (d, J = 8.2 Hz, 2H), 7.56 (d, J = 7.5 Hz, 1H), 7.38 (d, J = 7.8 Hz, 2H), 4.16 (q, J = 7.2 Hz, 2H), 3.04 (t, J = 7.8 Hz, 2H), 2.69 (t, 2H), 1.26 (t, J = 7.1 Hz, 3H).

**Compound 4**: A mixture of compound 2 (336.5 mg, 0.72 mmol), Compound 3 (235 mg, 0.6 mmol), Pd(PPh_3_)_4_ (34.7 mg, 0.03 mmol), and potassium carbonate (124.4 mg, 0.9 mmol) in THF (21 mL) and water (3 mL) was heated to reflux overnight. After cooling to room temperature, the mixture was extracted with CH_2_Cl_2_, and the combined organic layer was dried over anhydrous Na_2_SO_4_. After the solvent was evaporated, the residue was purified by column chromatography (silica gel, petroleum ether: CH_2_Cl_2_ = 1:1 as the eluent) to afford an orange-yellow solid in 80% yield (312 mg). ^1^H NMR (600 MHz, CDCl_3_): δ 7.89 (d, J = 8.2 Hz, 2H), 7.79 (d, J = 1.5 Hz, 1H), 7.75 (d, J = 7.3 Hz, 1H), 7.57 – 7.52 (m, 1H), 7.40 (d, J = 8.1 Hz, 2H), 7.06 – 7.04 (m, 4H), 6.87 – 6.84 (m, 5H), 4.16 (q, J = 7.1 Hz, 2H), 3.81 (s, 6H), 3.05 (t, J = 7.9 Hz, 2H), 2.70 (t, J = 7.9 Hz, 2H), 1.27 (t, J = 7.1 Hz, 3H).

**2FMPA-BT-PPA**: Compound 4 (195.5 mg, 0.3 mmol), KOH (168.3 mg, 3 mmol), THF (10 mL), and MeOH (2 mL) and heated to reflux for 10 h. After removing all solvent, the water was added, followed by the addition of 2M HCl (aq.) to precipitate the product. The product was further purified by flash column chromatography (silica gel, CH_2_Cl_2_: methanol = 20:1 as the eluent) to afford an orange solid in a yield of 92% (172 mg). ^1^H NMR (400 MHz, DMSO-d6): δ 7.90 (d, J = 7.8 Hz, 2H), 7.83 – 7.72 (m, 2H), 7.61 – 7.50 (m, 1H), 7.41 (d, J = 7.9 Hz, 2H), 7.05 (d, 4H), 6.86 (m, 5H), 3.81 (s, 6H), 3.07 (t, J = 7.7 Hz, 2H), 2.78 (t, J = 7.8 Hz, 2H). ^19^F NMR (376 MHz, DMSO-d6): δ -117.72 (s, 1F), δ -123.94 (s, 1F). ^13^C NMR (101 MHz, DMSO-d6) δ 174.16, 157.33, 156.21, 154.90, 153.87, 153.46, 141.90, 140.41, 136.92, 134.89, 133.45, 130.74, 129.59, 128.98, 128.04, 126.05, 125.19, 119.84, 115.34, 112.69, 55.76, 35.52, 30.61. HRMS: C_35_H_27_O_4_N_3_F_2_S calcd: 623.1690 found: 623.16635 [M]+.

**Figure S1**. ^1^H NMR spectrum of Compound 1 in CDCl_3_.

**Figure S2**. ^1^H NMR spectrum of Compound 2 in CDCl_3_.

**Figure S3**. ^1^H NMR spectrum of Compound 3 in CDCl_3_.

**Figure S4**. ^1^H NMR spectrum of Compound 4 in CDCl_3_.

**Figure S5**. ^1^H NMR spectrum of 2FMPA-BT-PPA in DMSO-d6.

**Figure S6**. ^19^F NMR spectrum of 2FMPA-BT-PPA in DMSO-d6.

**Figure S7**. ^13^C NMR spectrum of 2FMPA-BT-PPA in DMSO-d6.

**Figure S8**. HRMS spectrum of 2FMPA-BT-PPA.

**Computation method (Note S2)**

The binding energy was calculated within the framework of density functional theory (DFT) using the CP2K software package^1^, with the Perdew–Burke–Ernzerhof (PBE) exchange–correlation functional employed for all computations^2^. For model construction, the (111) surface of In_2_O_3_ was selected as the substrate for organic molecule adsorption. During the calculations, the bottom two atomic layers of the In_2_O_3_ (111) surface were fixed to mimic the bulk phase characteristics and eliminate spurious surface relaxation from the bulk region. A vacuum layer of 20 Å was introduced to avoid interactions between periodic images of the surface system. Key computational parameters were configured as follows: a plane-wave cutoff energy of 500 eV, an energy convergence threshold of 1×10-5 eV, and a maximum residual force criterion of 0.02 eV·Å^-1^.

Geometry optimizations were performed until full convergence was achieved, with the convergence strictly governed by the above energy and force thresholds. Based on the fully optimized structural models, ab initio molecular dynamics (AIMD) simulations were further performed^3,4^. The simulations were conducted under the NVT ensemble, with a Nose–Hoover thermostat employed to maintain a constant temperature of 300 K. A time step of 1 fs was adopted, and the total simulation duration was set to 10 ps—a timescale sufficient to capture the system’s dynamic relaxation process and evaluate its dynamic stability. To balance the reliability of calculation results and computational efficiency, a Γ-centered 1×1×1 k-point grid was used for the AIMD simulations.

Simulation model limitations and rationale:

All molecular dynamics simulations were performed on the idealized In_2_O_3_ (111) surface, the most thermodynamically stable and dominant exposed crystal plane of indium tin oxide (ITO). Although practical PEN/ITO substrates exhibit microscale roughness and heterogeneity, SAMs have dimensions of ~1–2 nm. At this nanoscale, local adsorption regions on even rough surfaces are sufficiently flat and homogeneous for molecular assembly, justifying the use of idealized single-crystal surfaces as a standard approximation in SAM simulation studies.

The primary purpose of these simulations was to compare the relative assembly freedom and conformational adjustability of PPA and BA molecules under identical substrate conditions, rather than to reproduce the absolute structure of the entire PEN/ITO interface. The observed differences in packing behavior between the two molecules arise from their intrinsic molecular structures and are not affected by the minor simplifications of the substrate model.

Notably, these simulations model the molecular self-assembly process on undeformed PEN/ITO substrates during device fabrication. The mechanical deformability of PEN/ITO substrates, which is relevant only to subsequent flexible device operation, does not influence the initial formation of SAMs and was therefore not considered in the simulation setup.

We acknowledge that idealized single-crystal surfaces cannot fully capture all complexities of real device interfaces; however, the model provides a valid and representative framework for investigating the fundamental differences in molecular assembly behavior between PPA and BA.

**Figure S9**. The C1s spectra of PEN/ITO/2FMPA-BT-BA (a) and PEN/ITO/2FMPA-BT-PPA (b); the In3d spectra of PEN/ITO/2FMPA-BT-BA (a) and PEN/ITO/2FMPA-BT-PPA (b).

**Figure S10**. The EDX spectroscopy mapping results of PEN/ITO/2FMPA-BT-BA (a) and PEN/ITO/2FMPA-BT-PPA (b).

**Figure S11**. The potential mapping of PEN/ITO/2FMPA-BT-BA (a) and PEN/ITO/2FMPA-BT-PPA (b) measured by KPFM. The area of the tested region is 3 μm×3 μm.

**Figure S12**. The surface morphology of PEN/ITO/2FMPA-BT-BA (a) and PEN/ITO/2FMPA-BT-PPA (b) measured by atomic force microscope. The area of the tested region is 3 μm×3 μm.

**Figure S13**. The SEM image of PEN/ITO/2FMPA-BT-BA (left) and PEN/ITO/2FMPA-BT-PPA (right).

**Figure S14**. PEN/ITO/SAMs/perovskite samples’ lateral force-normal force curves in nano-scratch measurements.

**Figure S15**. (a) and (b), the contour plot of transient absorption (TA) spectroscopy of ITO/BA/perovskite and ITO/PPA/perovskite (b); (c) and (d), The corresponding TA spectra at different decay times of ITO/BA/perovskite (c) and ITO/PPA/perovskite (d).

**Figure S16**. Time-resolved photoluminescence of the PEN/ITO/SAMs sample. The excitation is from the PEN/ITO side.

**Figure S17**. The PL spectra of PEN/ITO/SAMs/perovskite samples. The excitation is from the PEN/ITO side.

**Figure S18**. Density functional theory (DFT) calculated frontier molecular orbital distributions of BA and PPA molecules: (a) HOMO of BA, (b) HOMO of PPA, (c) LUMO of BA, (d) LUMO of PPA. Calculation was done with Gaussian09 package at a level of B3LYP/6-311G(d,p).

**Figure S19**. (a), The absorption spectrum of perovskite films deposited on ITO/2PACz and ITO/PhT-2PACz; (b) The corresponding Tauc plots.

**Figure S20**. The UPS measurements of the buried interface of perovskite film deposited on PEN/ITO/BA and PEN/ITO/PPA for determining the WF and VBM.

**Figure S21**. The energy level alignment at the ITO/SAMs/perovskite interface.

**Figure S22**. The PLQY measurements on perovskite films peeled off from PEN/ITO/BA/perovskite and PEN/ITO/PPA/perovskite.

**Figure S23**. The space-charge-limited current measurements for the hole-only device with a structure of PEN/ITO/SAMs/perovskite/PTAA/Ag.

**Figure S24**. GIXRD diffraction patterns of the bottom surface of perovskite films peeled from PEN/ITO/2FMPA-BT-BA/perovskite sample (a) and PEN/ITO/2FMPA-BT-PPA/perovskite sample (b) without any mechanical aging, with instrument angles from 0° to 40°.

**Figure S25**. The EQE spectra of the f-PSCs.

**Figure S26**. The stable power output of the f-PSCs.

**Figure S27**. The statistical photovoltaics performance for f-PSCs using 2FMPA-BT-BA and 2FMPA-BT-PPA.

**Figure S28**. The TPV (a) and TPC (b) measurements on f-PSCs.

**Figure S29**. The J–V curves of the f-PSCs under reverse and forward voltage scans (device area ~ 0.08 cm^-2^).

**Figure S30**. The champion J–V curves of the rigid PSCs using 2FMPA-BT-BA or 2FMPA-BT-PPA SAMs under reverse scan.

**Figure S31**. Normalized UV-absorption spectra of FMPA-BT-BA (left) and FMPA-BT-PPA (right) in solution state, film state on glass and film state on PEN/ITO.

**Figure S32**. The champion J-V curves of f-PSCs fabricated with FMPA-BT-BA and FMPA-BT-PPA. The average photovoltaic parameters for FMPA-BT-BA-based f-PSCs and FMPA-BT-PPBA-based f-PSCs, PCE: 24.01 ± 0.24%/22.62 ± 0.16%, V_OC_: 1.164 ± 0.004 V/1.158 ± 0.003 V, FF: 83.63 ± 0.42%/80.89 ± 0.79% and J_SC_: 24.14 ± 0.0.15 mA.cm^-2^/24.65 ± 0.0.19 mA.cm^-2^.

**Figure S33**. The schematic diagram of the cyclic bending test on f-PSCs using our home-built cyclic bending tester. Tester’s cyclic bending function is realized by a program-controlled stepper motor that drives a sliding block on a linear guide rail to perform reciprocating motion. Specifically, one end of the hinged sample clamp is fixed, while the other end is connected to the sliding block. The reciprocating motion of the sliding block drives the device mounted on the clamp to undergo cyclic bending. The process of one complete bending cycle: initially, the device is in a nearly flat state; the stepper motor then pushes one end of the sample clamp to the left to bend the device; finally, the stepper motor pulls the clamp back to the initial flat state, completing one bending cycle.

**Figure S34**. (a) The champion J-V curves of f-PSCs fabricated with MPA-BT-BA SAMs and MPA-EBT-BA SAMs. (b) The champion J-V curves of f-PSCs in (a) after 2000 outward bending cycles at a bending curvature radius of 3 mm.

**Figure S35**. The maximum power point tracking (MPPT) test of the encapsulated f-PSCs.

**Table S1**. Elemental quantification results for PEN/ITO/2FMPA-BT-BA obtained from EDX results

| Element | Line Type | Apparent concentration | k ratio | Wt% | Atomic% |
| --- | --- | --- | --- | --- | --- |
| C | K series | 3.75 | 0.03745 | 10.76 | 48.25 |
| N | K series | 3.00 | 0.00535 | 2.30 | 8.83 |
| F | K series | 0.29 | 0.00057 | 0.89 | 2.54 |
| S | K series | 0.00 | 0.00000 | 0.00 | 0.00 |
| In | L series | 54.95 | 0.49462 | 86.05 | 40.38 |
| Total |  |  |  | 100.00 | 100.00 |

**Table S2**. Elemental quantification results for PEN/ITO/2FMPA-BT-PPA obtained from EDX results

| Element | Line Type | Apparent concentration | k ratio | Wt% | Atomic% |
| --- | --- | --- | --- | --- | --- |
| C | K series | 4.63 | 0.04626 | 12.28 | 50.64 |
| N | K series | 3.49 | 0.00621 | 2.61 | 9.24 |
| F | K series | 0.56 | 0.00109 | 1.56 | 4.08 |
| S | K series | 0.00 | 0.00000 | 0.00 | 0.00 |
| In | L series | 57.20 | 0.51485 | 83.54 | 36.04 |
| Total |  |  |  | 100.00 | 100.00 |

**Table S3**. The averaged photovoltaics performance of rigid PSCs.

|  | PCE (%) | V_OC_ (V) | J_SC_ (mA.cm^-2^) | | FF (%) |
| --- | --- | --- | --- | --- | --- |
| 2FMPA-BT-BA | 24.02 ± 0.36 | 1.158 ± 0.007 | 25.16 ± 0.21 | 82.46 ± 0.62 | |
| 2FMPA-BT-PPA | 25.77 ± 0.40 | 1.179 ± 0.007 | 25.81 ± 0.16 | 84.61 ± 1.13 | |

Note: the performance parameters in the table are the average values of the five devices.

Table S4. The recently reported efficient SAMs-based f-PSCs and their mechanical robustness^5-16^.

| PCE (%) | V_OC_ (V) | FF (%) | V_OC_ × FF | Mechanical durability | Reference | |
| --- | --- | --- | --- | --- | --- | --- |
| 25.05 | 1.186 | 83.14 | 0.986 | 5 mm, 5000 cycles, 93% | Adv. Mater., 2024, 36(38): 2407032 | |
| 25.13 | 1.121 | 82.03 | 0.992 | 10 mm, 5000 cycles, 95% | Angew. Chem.-Int. Edit., 2025, 64(24): e202501267 | |
| 24.75 | 1.174 | 84.65 | 0.993 | 3 mm, 10000 cycles, 96.5% | Energy Environ. Sci., 2025, 18(19): 8803-8814 | |
| 24.48 | 1.174 | 81.74 | 0.959 | 5 mm, 10000 cycles, 80% | Energy Environ. Sci., 2025, 18(9): 4324-4334 | |
| 24.03 | 1.185 | 81.70 | 0.968 | 8 mm, 5000 cycles, 90% | Energy Environ. Sci., 2025, 18(5): 2436-2451 | |
| 24.68 | 1.159 | 86.07 | 0.997 | 5 mm, 1000 cycles, 96% | Energy Environ. Sci., 2025, 18(6): 2905-2917 | |
| 24.64 | 1.166 | 83.40 | 0.972 | 5 mm, 10000 cycles, 81% | Sci. Adv., 2025, 11(5): eadr2290 | |
| 24.19 | 1.165 | 82.66 | 0.963 | 4 mm, 1000 cycles, 92% | Nano Lett., 2025, 25(42): 15249-15255 | |
| 24.16 | 1.167 | 83.00 | 0.968 | 4 mm, 1000 cycles, 92% | Adv. Mater., 2026, 38(7): e19365 | |
| 25.19 | 1.180 | 84.25 | 0.994 | 3 mm, 5000 cycles, 97% | ACS nano, 2025, 19(31): 28888-28899 | |
| 24.95 | 1.167 | 85.60 | 0.999 | 5 mm, 10000 cycles, 90% | Energy Environ. Sci.,  2026, 19, 1983 | |
| 26.22 | 1.196 | 84.30 | 1.008 | 3 mm, 6000 cycles, 90% | Nat. Synth., 2026, 5(2): 209-220 | |
| **25.26** | **1.180** | **85.16** | **1.005** | **3 mm, 10000 cycles, 98%** | | **This work** |

Note: For “Mechanical durability” column, take “5 mm, 5000 cycles, 93%” as an example, this means the corresponding devices maintained 93% of their initial PCE after 5000 bending cycles at a bending radius of 5 mm.

**References**

1.	Kühne T D, Iannuzzi M, Del Ben M, et al. CP2K: An electronic structure and molecular dynamics software package-Quickstep: Efficient and accurate electronic structure calculations[J]. The Journal of Chemical Physics, 2020, 152(19).

2.	Grimme S, Ehrlich S, Goerigk L. Effect of the damping function in dispersion corrected density functional theory[J]. Journal of computational chemistry, 2011, 32(7): 1456-1465.

3.	Martyna G J, Klein M L, Tuckerman M. Nosé–Hoover chains: The canonical ensemble via continuous dynamics[J]. The Journal of chemical physics, 1992, 97(4): 2635-2643.

4.	Tuckerman M, Berne B J, Martyna G J. Reversible multiple time scale molecular dynamics[J]. The Journal of chemical physics, 1992, 97(3): 1990-2001.

5. Tong X, Xie L, Li J, et al. Large orientation angle buried substrate enables efficient flexible perovskite solar cells and modules[J]. Advanced Materials, 2024, 36(38): 2407032.

6. Liang H, Zhu W, Lin Z, et al. Enhancing Efficiency and Stability of Inverted Flexible Perovskite Solar Cells via Multi‐Functionalized Molecular Design[J]. Angewandte Chemie International Edition, 2025, 64(24): e202501267.

7. Zhou B, Li M, Xiong Q, et al. Soft conjugation extension strategy of self-assembled molecules for achieving efficient and mechanically stable flexible perovskite solar cells[J]. Energy & Environmental Science, 2025, 18(19): 8803-8814.

8. Xu Z, Yu R, Xue T, et al. Stress release via thermodynamic regulation towards efficient flexible perovskite solar cells[J]. Energy & Environmental Science, 2025, 18(9): 4324-4334.

9. Luo S, Cao S, Zheng T, et al. Melamine holding PbI 2 with three “arms”: an effective chelation strategy to control the lead iodide to perovskite conversion for inverted perovskite solar cells[J]. Energy & Environmental Science, 2025, 18(5): 2436-2451.

10. Fan X, Chen J, Wang J, et al. Efficient rigid and flexible perovskite solar cells using strongly adsorbed molecules for lattice repair and grain boundary mitigation[J]. Energy & Environmental Science, 2025, 18(6): 2905-2917.

11. Zhang W, Liu J, Song W, et al. Chemical passivation and grain-boundary manipulation via in situ cross-linking strategy for scalable flexible perovskite solar cells[J]. Science Advances, 2025, 11(5): eadr2290.

12. Wei F, Wang J, Wang T, et al. Efficient Rigid and Flexible Perovskite Solar Cells with Uniform Crystallization and Weak Lattice Vibration via Interface Defect Passivation[J]. Nano Letters, 2025, 25(42): 15249-15255.

13. Sun X, Gong J, Liu Q, et al. Molecular Tailoring of Self‐Assembled Monolayers via Polar Ether Linker for Highly Efficient and Mechanically Robust Flexible Perovskite Solar Cells[J]. Advanced Materials, 2026, 38(7): e19365.

14. Gao S, Fan X, Wang J, et al. Over 26% and 24%-Efficiency Rigid and Flexible Perovskite Photovoltaics through Enhanced Crystallinity and Alleviated Residual Strains[J]. ACS nano, 2025, 19(31): 28888-28899.

15. Xing C, Zhang W, Zhou H, et al. Exploring a Scalable Route for Efficient Flexible Perovskite Solar Cells via Amphiphilic Cross-linkable Monomer[J]. Energy & Environmental Science, 2026, 19, 1983.

16. Shi S, Gong C, Tao M, et al. Mechanically interlocked polymer scaffolds enable high-efficiency printed flexible perovskite photovoltaics[J]. Nature Synthesis, 2026, 5(2): 209-220.
